# Testis‐Enriched F‐Box Protein FBXO39 Is Important for Spermiogenesis and Male Fertility in Mice

**DOI:** 10.1111/andr.70225

**Published:** 2026-03-31

**Authors:** Yuki Kaneda, Haruhiko Miyata, Masahito Ikawa

**Affiliations:** ^1^ Research Institute for Microbial Diseases The University of Osaka Suita Osaka Japan; ^2^ Graduate School of Pharmaceutical Sciences The University of Osaka Suita Osaka Japan; ^3^ The Institute of Medical Science The University of Tokyo Minato‐ku Tokyo Japan; ^4^ Center for Infectious Disease Education and Research The University of Osaka Suita Osaka Japan; ^5^ Center for Advanced Modalities and DDS (CAMaD) The University of Osaka Suita Osaka Japan

**Keywords:** F‐box proteins, male fertility, spermatogenesis

## Abstract

**Background:**

The SCF (Skp–Cullin–F‐box) complex is a major class of E3 ubiquitin ligases. F‐box proteins constitute the SCF complex and play a critical role in recognizing substrates for ubiquitination. In mice, several F‐box proteins, including FBXO36 and FBXO39, are predominantly expressed in testes.

**Objective:**

To investigate the roles of FBXO36 and FBXO39 in mouse male reproduction.

**Materials and Methods:**

We generated *Fbxo36* KO and *Fbxo39* KO mouse lines using the CRISPR/Cas9 system. We evaluated male fertility, sections of reproductive organs, and sperm morphology and motility.

**Results:**

No overt abnormalities were found in male fertility or sperm morphology of *Fbxo36* KO mice. In contrast, *Fbxo39* KO males showed subfertility, abnormal sperm morphology, and reduced sperm motility. Histological analyses of testis sections revealed that FBXO39 is critical for the morphological transformation of round spermatids into spermatozoa.

**Discussion and Conclusion:**

Our findings demonstrate that *Fbxo39* is important for spermiogenesis in mice, whereas *Fbxo36* is dispensable for male fertility. Further analyses of FBXO39 may contribute to understanding the etiology of male infertility.

## 1 Introduction

Spermatozoa are produced through a specialized process called spermatogenesis, which takes place within the seminiferous tubules of the testes. This complex process proceeds through a series of stages. Initially, spermatogonia divide by mitosis to give rise to spermatocytes. These spermatocytes then undergo meiosis, reducing their chromosome number and producing haploid round spermatids. Spermiogenesis is the final phase of spermatogenesis, during which haploid round spermatids undergo extensive morphological and functional remodeling to produce spermatozoa. This process involves nuclear condensation through chromatin remodeling, acrosome biogenesis, formation of the flagellum with axonemal and accessory structures, and removal of excess cytoplasm during spermiation [1, 2]. Each step is tightly coordinated, and defects at any stage could compromise sperm structure and function, ultimately leading to male infertility [1].

The ubiquitin–proteasome system (UPS) is a major pathway for the degradation of intracellular proteins. Proteins destined for degradation are tagged with ubiquitin and subsequently processed by the 26S proteasome [3, 4]. This pathway is crucial for cellular homeostasis and regulates diverse biological processes such as cell cycle progression, signal transduction, and differentiation [3, 5, 6, 7]. A key determinant of substrate specificity in the UPS is the E3 ubiquitin ligase. Among them, the SCF (SKP1–Cullin–F‐box) complex represents a prototypical E3 ligase [8]. Within the SCF complex, F‐box proteins act as substrate‐recognition subunits, conferring precise and timely degradation of target proteins [8].

F‐box proteins can be classified into three subfamilies based on their C‐terminal domains: FBXL (containing leucine‐rich repeats), FBXW (containing WD40 repeats), and FBXO (containing other or uncharacterized domains) [9]. Several FBXO proteins are highly expressed in testes, suggesting their specialized functions in spermatogenesis. Functional studies have revealed roles of some FBXO proteins using knockout (KO) mouse models; *Fbxo24*, *Fbxo43*, and *Fbxo47* are essential for male fertility [10, 11, 12, 13, 14, 15] while *Fbxo15* and *Fbxo30* are not [16, 17]. For example, we previously demonstrated that loss of *Fbxo24* causes severe defects in sperm flagellar formation in mice [11]. However, there remain testis‐enriched *Fbxo* genes whose physiological functions have yet to be elucidated.

In this study, we analyzed *Fbxo36* and *Fbxo39*, which are predominantly expressed in the testis but remain uncharacterized with KO mice. We generated *Fbxo36* and *Fbxo39* KO mice, and assessed male fertility, sperm morphology and motility, and histology of reproductive organs. Our analyses revealed that *Fbxo36* is dispensable for male fertility, whereas *Fbxo39* is critical for spermiogenesis.

## 2 Material and Methods

### 2.1 Animals

All animal experiments were approved by the Animal Care and Use Committee of the Research Institute for Microbial Diseases, Osaka University (#Biken‐AP‐H30‐01 and #Biken‐AP‐R03‐01). Mice were purchased from CLEA Japan (Tokyo, Japan) or Japan SLC (Shizuoka, Japan) and maintained under 12‐h light/dark cycles with ad libitum access to food and water. Wild‐type or heterozygous mice were used as controls. Frozen spermatozoa from all gene‐modified mice generated in this study will be made available through the RIKEN BioResource Research Center or the Center for Animal Resources and Development (CARD), Kumamoto University.

### 2.2 Generation of KO Mice

Both *Fbxo36* and *Fbxo39* KO mouse lines were established as previously described [11, 18]. Briefly, crRNAs were annealed with tracrRNA (#TRACRRNA05N‐5NMOL, Sigma‐Aldrich) and combined with Cas9 (A36498, Thermo Fisher Scientific) to form CRISPR/Cas9 ribonucleoprotein complexes at 37°C for 5 min. The complexes were delivered into fertilized eggs (B6D2F1 x B6D2F1) via electroporation using the NEPA21 Super Electroporator (NEPA GENE, Chiba, Japan). Two‐cell‐stage embryos cultured in KSOM medium were transferred into pseudopregnant ICR females. Offspring were genotyped by PCR and confirmed by Sanger sequencing. Used gRNA sequences are shown in Table S1.

### 2.3 Phylogenetic Analysis

Phylogenetic relationships of mouse testis–enriched FBXO proteins were analyzed as previously described [19] using the Neighbor‐Joining method implemented in MEGA11 [20].

### 2.4 In Silico Analysis of Gene Expression

Transcriptome data from various mouse tissues were obtained from a previous study [21], and average gene expression values were processed and normalized using Z‐scores as described previously [19]. Single‐cell transcriptome data from mouse and human testes were obtained [22] and analyzed using Loupe Cell Browser 3.3.1 (10X Genomics, Pleasanton, CA, USA).

### 2.5 Domain Analysis and Structural Prediction

Amino acid sequences were downloaded from the Consensus CDS (CCDS) database (https://www.ncbi.nlm.nih.gov/projects/CCDS/CcdsBrowse.cgi). The following sequences were used in the analysis: mouse FBXO36 (CCDS35634.1), human FBXO36 (CCDS2472.1), mouse FBXO39 (CCDS36214.1), human FBXO39 (CCDS11082.1), mouse RBX1 (CCDS49676.1), mouse CUL1 (CCDS20095.1), and mouse SKP1 (CCDS24669.1). Protein domain was predicted using Simple Modular Architecture Research Tool (SMART) (https://smart.embl.de/) [23]. Structural models were generated using the AlphaFold3 server (https://deepmind.google/science/alphafold/alphafold‐server/) [24].

### 2.5 Plasmids and Cell Transfection

Plasmids were constructed as previously reported [11]. The open reading frames (ORFs) of *Fbxo36* and *Fbxo39* were amplified from mouse testis cDNA and inserted into the multiple cloning site of pCAG1.1 vectors (Addgene, Plasmid #173685) containing various epitope tags. ΔF‐box mutant constructs were generated via inverse PCR using the KOD ‐Plus‐ Mutagenesis Kit (Toyobo, Tokyo, Japan) following the manufacturer's instructions. Primer sequences used for cloning are shown in Table S1. The pCAG1.1‐*Skp1*‐1D4 plasmid was obtained from our previous work [11]. Plasmid DNA was transfected into HEK293T cells using the calcium phosphate method as described in [25].

### 2.6 Reverse Transcription Polymerase Chain Reaction (RT‐PCR)

RT‐PCR was carried out as previously reported [26]. Adult mouse multi‐tissue samples were collected from C57BL/6N mice. Total RNA was extracted and purified using TRIzol reagent (Thermo Fisher Scientific, Waltham, MA, USA). First‐strand cDNA was synthesized with SuperScript IV (Thermo Fisher Scientific) using oligo(dT) primers. PCR amplification was performed using KOD Fx Neo DNA Polymerase (Toyobo, Tokyo, Japan). Primer sequences are shown in Table S1.

### 2.7 Histological Analysis of Testis and Epididymis

Periodic acid–Schiff (PAS) and hematoxylin staining were performed as previously reported [26]. Testes and cauda epididymides were fixed in Bouin's solution (Polysciences, Inc., PA, USA), embedded in paraffin, sectioned, rehydrated, and treated with 1% periodic acid for 10 min, followed by Schiff's reagent (FUJIFILM Wako, Osaka, Japan) for 20 min. Sections were counterstained with Mayer's hematoxylin solution (FUJIFILM Wako) and examined using an Olympus BX53 microscope equipped with a DP74 color camera (Olympus, Tokyo, Japan).

### 2.8 Analysis of Sperm Morphology and Motility

Spermatozoa were collected from the cauda epididymis and suspended in TYH medium [27]. Sperm morphological analysis was performed as previously reported [18]. Sperm motility was evaluated as previously reported [11] using the CEROS II system (software version 1.5; Hamilton Thorne Biosciences, MA, USA) after incubation for 10 and 120 min in TYH medium. The 10‐min time point was selected to assess initial motility, taking into account the time required for sample preparation, whereas the 120‐min time point was used to evaluate motility after capacitation [28]. Progressive motility was defined as VSL/VAP ≥ 0.8 and VAP ≥ 50 µm/s.

### 2.9 Immunostaining of Cryo‐Sections of Cauda Epididymis

Immunostaining of cauda epididymal cross‐sections was performed as previously reported [11]. Briefly, cauda epididymides were fixed in 4% paraformaldehyde in PBS for 3 h at 4°C, followed by infiltration with 15% and 30% sucrose in PBS at 4°C. The tissues were embedded in OCT compound (Tissue‐Tek, Miami, FL, USA), frozen in liquid nitrogen, and sectioned at 10 µm thickness using a cryostat. Used antibodies are shown in Table S2.

### 2.10 Immunoblotting

Immunoblotting was performed as previously reported [18]. HEK293T cells were lysed in 1% Triton X‐100 lysis buffer [1% Triton X‐100, 50 mM Tris‐HCl pH 7.4, 150 mM NaCl, 1% (v/v) phosphatase inhibitor cocktail (Nacalai Tesque, Kyoto, Japan), 1% (v/v) protease inhibitor cocktail (Nacalai Tesque)] at 4°C with end‐over‐end rotation for 1 h. The supernatants were collected after centrifugation at 15,300 × *g* for 15 min at 4°C and subjected to SDS‐PAGE, followed by transfer to PVDF membranes and immunodetection. Used antibodies are shown in Table S2.

### 2.11 Co‐Immunoprecipitation

Co‐immunoprecipitation (Co‐IP) was performed as previously reported [18] with minor modifications. HEK293T cells were lysed in the same Triton X‐100 lysis buffer as described above and incubated at 4°C for 1 h with rotation. After centrifugation at 15,300 × *g* for 15 min at 4°C, the supernatants were collected. For antibody coupling, Dynabeads (#10009D, Thermo Fisher Scientific) were incubated with anti‐1D4 antibody (a kind gift from Dr. Martin M. Matzuk) at room temperature for 10 min. The antibody‐conjugated beads were then incubated with the supernatants for 1 h at 4°C. Immune complexes were eluted with 2× SDS sample buffer (132 mM Tris‐HCl, pH 6.8; 4% SDS; 20% glycerol; 0.01% bromophenol blue) for 10 min at 70°C and analyzed by immunoblotting.

### 2.12 Ultrastructural Analysis of Testis Using Transmission Electron Microscopy (TEM)

Ultrathin sections of testes and epididymis were prepared as previously reported [26, 29]. Briefly, testes and epididymis were fixed by perfusion and immersion in 4% paraformaldehyde for 6 h at 4°C and then cut into ∼2 mm^3^ blocks. Tissues were further fixed in 1% glutaraldehyde in HEPES buffer, post‐fixed with 1% osmium tetroxide and 0.5% potassium ferrocyanide, and rinsed with distilled water. Samples were dehydrated through a graded ethanol series (50%, 70%, and 90% on ice; 100% at room temperature), treated twice with 100% propylene oxide, and infiltrated with a propylene oxide–epoxy resin mixture before embedding in pure epoxy resin for 2 days at 60°C. Ultrathin sections (80 nm) were cut, stained with 2% uranyl acetate followed by lead solutions, with brief distilled water washes between staining steps. The sections were examined using a JEM‐1400 Plus transmission electron microscope (JEOL, Tokyo, Japan) operated at 80 kV, and images were acquired with a Veleta 2K × 2K CCD camera (Olympus, Tokyo, Japan).

### 2.14 Statistical Analyses

Statistical analyses were performed using a two‐tailed Welch's *t*‐test in Microsoft Excel (Microsoft Corporation, WA, USA). *p*‐values < 0.05, 0.01, and 0.001 were considered statistically significant and denoted as (*), (**), and (***), respectively. Error bars in all graphs indicate standard deviation.

## 3 Results

### 3.1 *Fbxo36* and *Fbxo39* Are Testis‐Enriched in Mice and Highly Conserved in Humans

**FIGURE 1:**
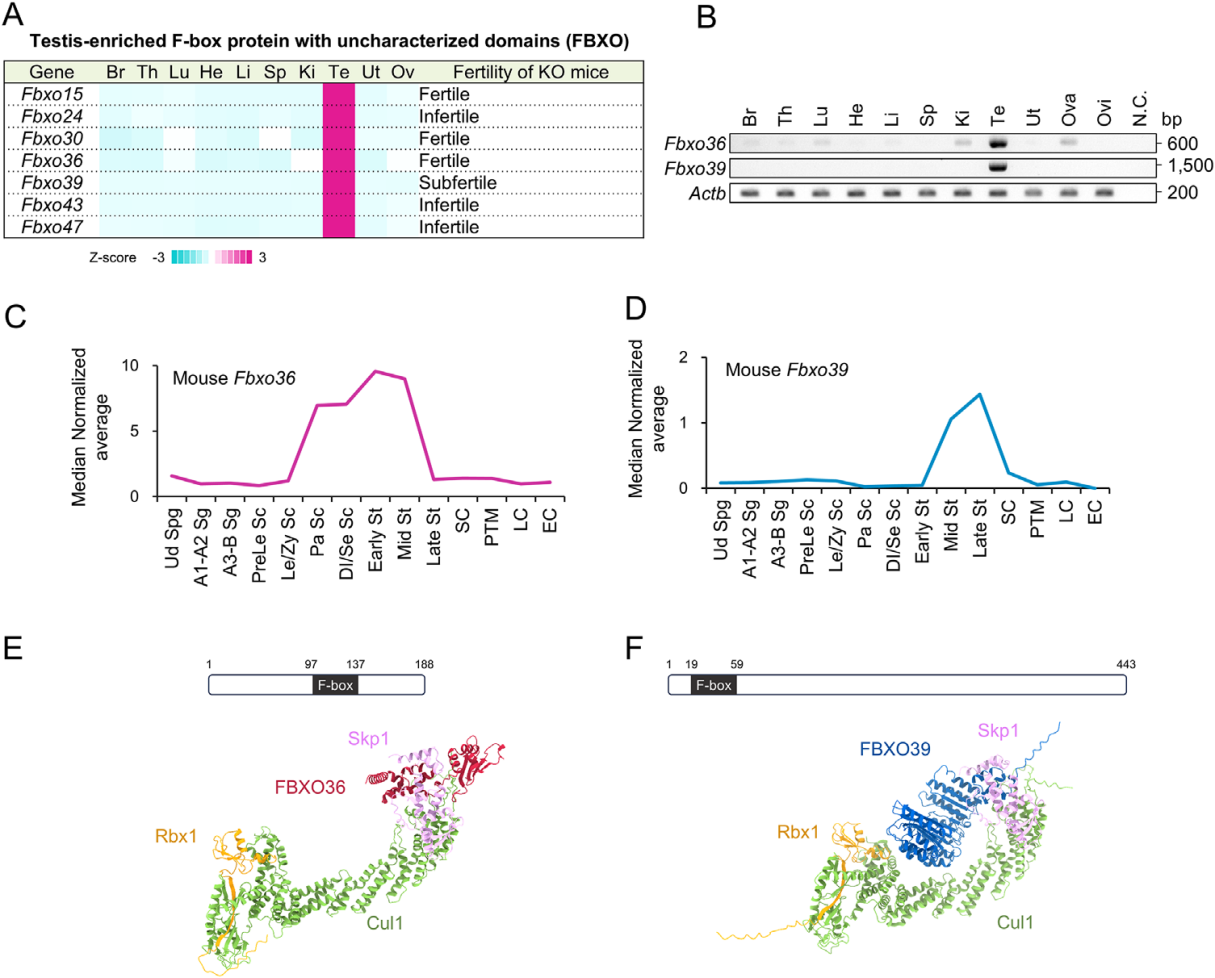
*Fbxo36* and *Fbxo39*, members of the FBXO family, are predominantly expressed in male germ cells. (A) List of testis‐enriched FBXO family members and their KO mouse fertility. FBXO proteins with a testis Z‐score more than 2.9 were selected. (B) RT‐PCR analyses of *Fbxo36* and *Fbxo39* using cDNA from multiple adult mouse tissues. Both *Fbxo36* and *Fbxo39* are predominantly expressed in the testis. Br: brain, Th: thymus, Lu: lung, He: heart, Li: liver, Sp: spleen, Ki: kidney, Te: testis, Ut: uterus, Ov: ovary, Ovi: oviduct, and N.C.: negative control (water). *Actb* was used as a loading control. (C) Expression of *Fbxo36* in each cell type in mouse testes. Cell types are abbreviated as follows: undifferentiated spermatogonia (Ud Spg); differentiating spermatogonia A1 and A2 (A1–A2 Spg); differentiating spermatogonia A3, A4, intermediate, and B (A3–B Spg); preleptotene spermatocytes (Prele Sc); leptotene/zygotene spermatocytes (Le/Zy Sc); pachytene spermatocytes (Pa Sc); diplotene and secondary spermatocytes (Di/Se Sc); early, mid, and late round spermatids (Early St, Mid St, and Late St); Sertoli cells (SC); peritubular myoid cells (PTM); Leydig cells (LC); and endothelial cells (EC). (D) Expression of *Fbxo39* in each cell type in mouse testes. (E) Predicted 3D structure of SCF (FBXO36) complex using AlphaFold3. The F‐box domain was detected using SMART. (F) Predicted 3D structure of SCF (FBXO39) complex using AlphaFold3. The F‐box domain was detected using SMART.

To identify testis‐enriched *Fbxo* genes that may play roles in spermatogenesis, we referred to transcriptomic analyses of adult mouse tissues reported by Li et al. [21]. Seven testis‐enriched *Fbxo* genes (*Fbxo15*, *Fbxo24*, *Fbxo30*, *Fbxo36*, *Fbxo39*, *Fbxo43*, and *Fbxo47*) were identified (Figure 1A). A phylogenetic analysis was performed to examine the evolutionary relationships of these mouse proteins (Figure S1A). Previous studies reported that *Fbxo24* is essential for spermiogenesis [10, 11, 12], and that *Fbxo43* and *Fbxo47* are required for the progression of meiotic prophase I [13, 14, 15]. In the present study, we focused on *Fbxo36* and *Fbxo39*, whose KO mouse phenotypes have not been reported.

RT‐PCR analyses of multiple mouse tissues confirmed that both *Fbxo36* and *Fbxo39* are predominantly expressed in the testis (Figure 1B). To examine evolutionary conservation between mice and humans, pairwise amino acid sequence alignments were performed using EMBOSS Needle (https://www.ebi.ac.uk/jdispatcher/psa/emboss_needle). FBXO36 and FBXO39 exhibited high sequence homology (76.1% identity for FBXO36; 83.7% identity for FBXO39), suggesting conserved functions between these species (Figure S1B, C). Given this high degree of sequence conservation, we next asked whether their expression patterns during spermatogenesis are also conserved. Single‐cell RNA‐seq analysis of mouse testes revealed stage‐specific expression patterns of *Fbxo36* and *Fbxo39*. *Fbxo36* expression increased from the pachytene spermatocyte stage, whereas *Fbxo39* expression became prominent from the mid‐spermatid stage (Figure 1C, D). Consistent with these findings, human *FBXO36* and *FBXO39* exhibited similar expression patterns in male germ cells (Figure S1D, E). These results, together with the high sequence conservation, suggest that the functions of FBXO36 and FBXO39 may be conserved between mice and humans.

F‐box proteins interact with SKP1 via their F‐box domain to form the SCF complex. Structural predictions using AlphaFold3 reliably modeled the SCF complexes for both FBXO36 and FBXO39 (Figures 1E, F and S2A, B), supporting their potential function as components of the E3 ubiquitin ligases.

### 3.2 Disruption of *Fbxo36* Did Not Impair Male Fertility in Mice

**FIGURE 2:**
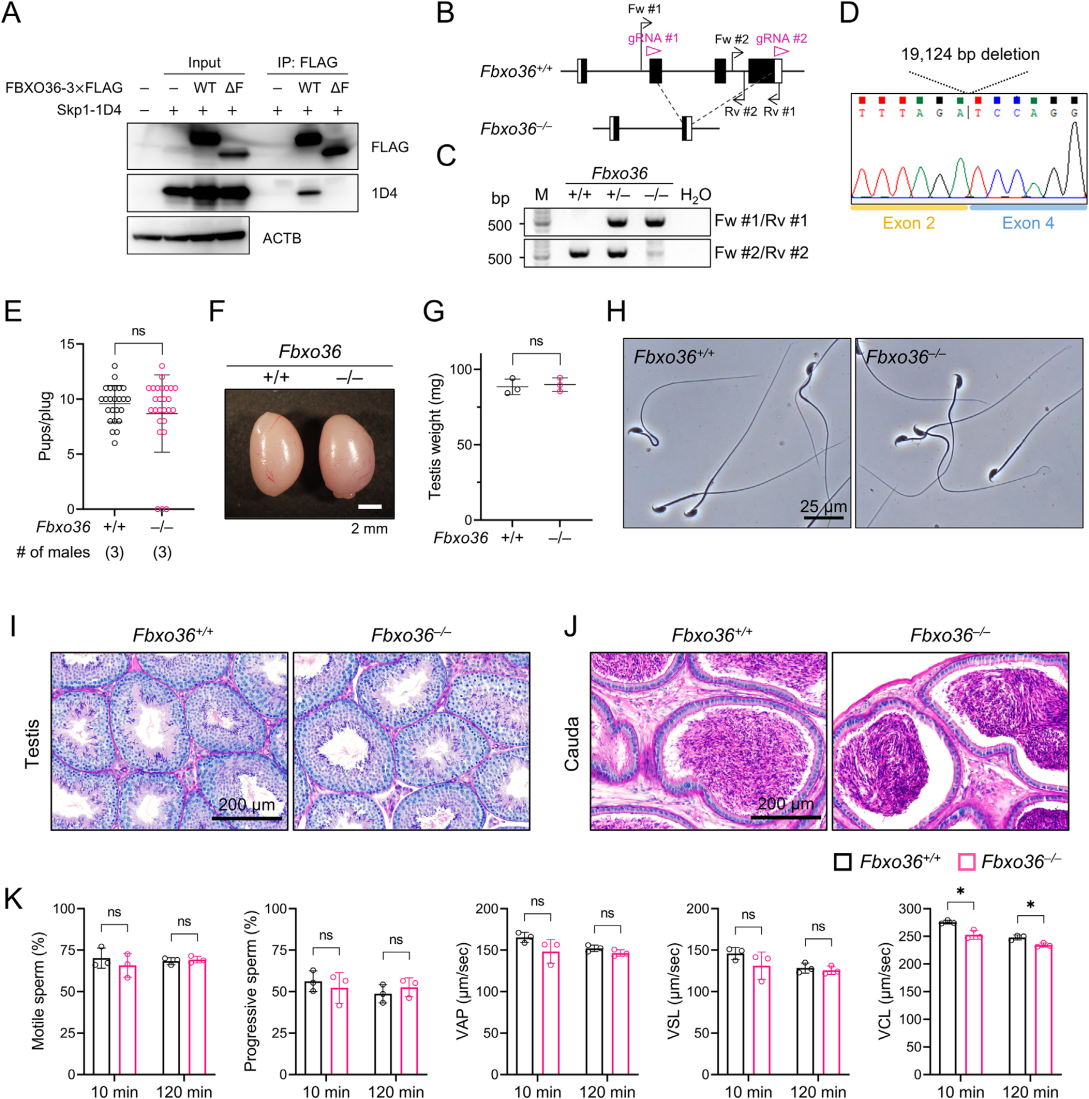
Phenotypic analysis of *Fbxo36* KO male mice. (A) HEK293T cells were transiently transfected with *Fbxo36 (WT)*‐3 × FLAG or *Fbxo36 (ΔF)*‐3× FLAG lacking the F‐box domain together with *Skp1*‐1D4. Co‐IP was carried out using an anti‐FLAG antibody. The wild‐type FBXO36 interacted with SKP1‐1D4, whereas the ΔF mutant lacked this interaction. ACTB served as the loading control. (B) Schematic representation of *Fbxo36* KO mouse generation using the CRISPR/Cas9 system. Untranslated regions are shown as white boxes, whereas coding sequences are indicated by black boxes. gRNAs are highlighted in pink. Forward (Fw) and reverse (Rv) primers used for genotyping are indicated. (C) Genotyping strategy for *Fbxo36* KO mice. As summarized in Table S1, primer pair Fw#1/Rv#1 was used to amplify the KO allele, whereas primer pair Fw#2/Rv#2 was used for the WT allele. (D) A deletion of 19,124 bp in the KO allele was confirmed by Sanger sequencing. (E) Pups per mating plug. WT or *Fbxo36* KO males were each paired with three WT females. (F) Gross morphology of the testes. (G) Testis weight measurements. (H) Morphological observation of cauda epididymal spermatozoa from WT and *Fbxo36* KO males. (I) Periodic acid–Schiff (PAS) and hematoxylin staining of testis sections. (J) PAS and hematoxylin staining of cauda epididymal sections. (K) Sperm motility analysis following incubation in capacitation medium. Percentages of motile and progressively motile spermatozoa were evaluated at 10 min and 120 min. Kinematic parameters, including average path velocity (VAP), straight‐line velocity (VSL), and curvilinear velocity (VCL), were also assessed at the same time points.

FBXO36 contains a conserved F‐box domain, which mediates interaction with SKP1 in the SCF complex. To examine whether FBXO36 associates with SKP1, HEK293T cells were transiently transfected with SKP1 together with either wild‐type FBXO36 or an F‐box–deleted FBXO36 (ΔF‐box). Co‐IP analysis showed that SKP1 bound to wild‐type FBXO36 but not to ΔF‐box FBXO36 (Figure 2A), suggesting that FBXO36 can associate with the SCF complex through its F‐box domain. To investigate the function of *Fbxo36* in vivo, we generated *Fbxo36* KO mice using the CRISPR/Cas9 system. Two guide RNAs were designed to introduce a large deletion in the *Fbxo36* ORF (Figure 2B). The Cas9–sgRNA ribonucleoprotein complex was delivered into 60 zygotes by electroporation. Fifty‐seven embryos were then transferred into the oviducts of 2 pseudopregnant females, and 13 pups were obtained, of which 7 carried large deletions. The founder mouse was crossed with wild‐type mice, and subsequent intercrossing of heterozygous offspring resulted in the generation of KO mice (Figure 2C). Sanger sequencing verified a 19,124 bp deletion (Figure 2D).

To assess fertility, each *Fbxo36* KO male was mated with three wild‐type (WT) females for 2 months. *Fbxo36* KO males did not exhibit a significant reduction in the number of pups per plug compared with WT controls (Figure 2E), indicating that *Fbxo36* is dispensable for male fertility. To further evaluate phenotypic differences, we analyzed multiple aspects of the male reproductive system. Testis morphology and weights were indistinguishable between the two genotypes (Figure 2F, G). No abnormalities were found in mature spermatozoa collected from *Fbxo36* KO cauda epididymis (Figure 2H). Further, periodic acid–Schiff (PAS) staining of testicular and epididymal cross‐sections revealed no apparent histological abnormalities (Figure 2I, J).

We also performed computer‐assisted sperm analysis (CASA) and found no significant differences in motility parameters in *Fbxo36* KO males, except for curvilinear velocity (VCL), which was modestly but significantly decreased (Figure 2K). Collectively, these phenotypic analyses demonstrate that disruption of *Fbxo36* does not cause marked impairments in spermatogenesis in mice.

### 3.3 Disruption of *Fbxo39* Results in Impaired Male Fertility and Defective Spermiogenesis in Mice

**FIGURE 3:**
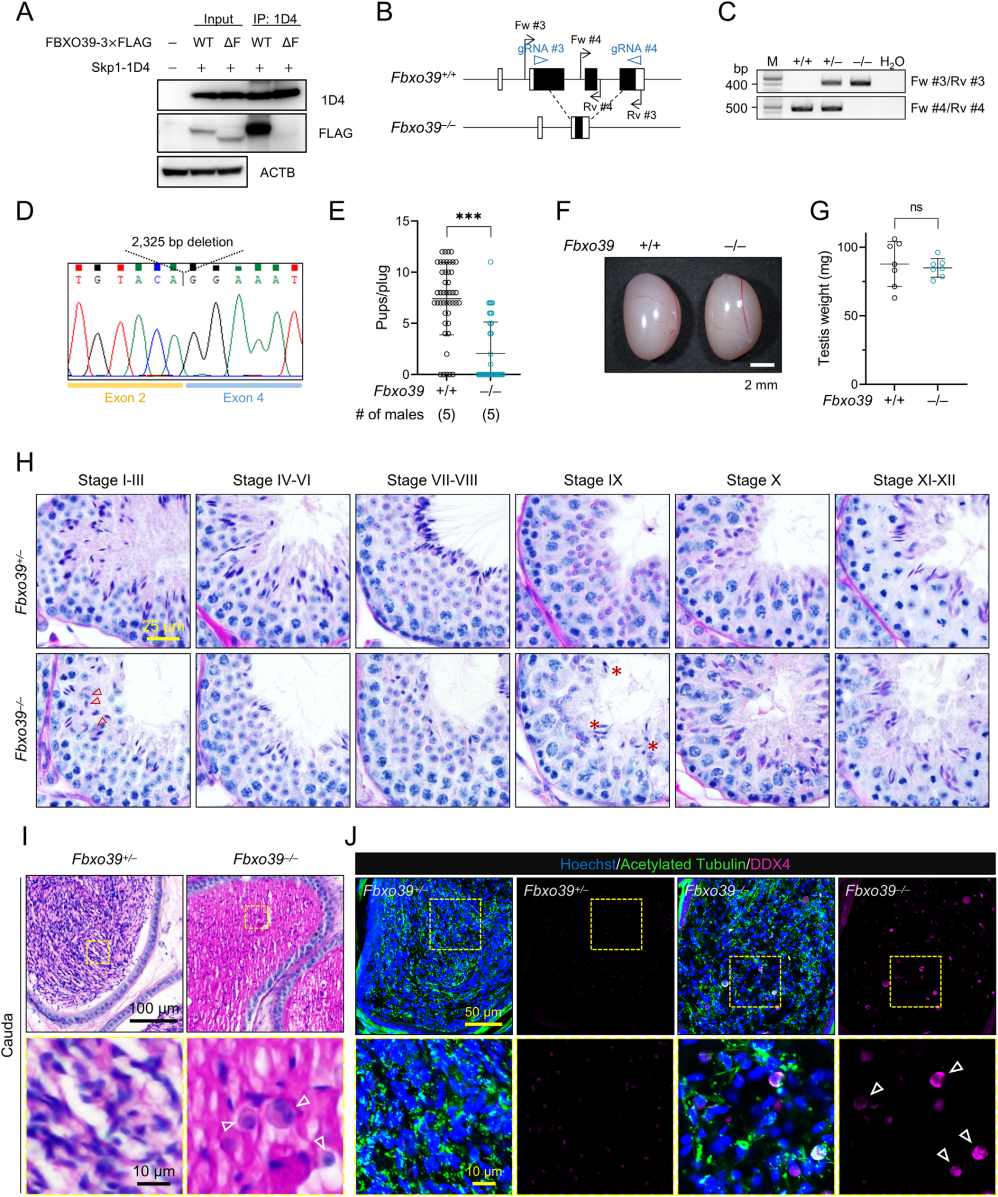
Generation of *Fbxo39* KO mice and histological analyses. (A) HEK293T cells were transiently transfected with *Fbxo39 (WT)*‐3× FLAG or *Fbxo39 (ΔF)*‐3× FLAG lacking the F‐box domain together with *Skp1*‐1D4. Co‐IP was carried out using an anti‐1D4 antibody. The wild‐type FBXO39 interacted with SKP1‐1D4, whereas the ΔF mutant lacked this interaction. ACTB served as the loading control. (B) Schematic representation of *Fbxo39* KO mouse generation using the CRISPR/Cas9 system. (C) Genotyping strategy for *Fbxo39* KO mice. As summarized in Table S1, primer pair Fw#3/Rv#3 was used to amplify the KO allele, whereas primer pair Fw#4/Rv#4 was used for the WT allele. (D) A deletion of 2,325 bp in the KO allele was confirmed by Sanger sequencing. (E) Pups per mating plug. WT or *Fbxo39* KO males were each paired with three WT females. (F) Gross morphology of the testes. (G) Testis weight measurements. (H) PAS and hematoxylin staining of the testis. Seminiferous tubules were categorized into stages I–III, IV–VI, VII–VIII, IX, X, and XI–XII. Red arrowheads indicate abnormally shaped sperm heads. Red asterisks highlight elongated spermatids that remained abnormally within tubules. (I) PAS and hematoxylin staining of cauda epididymal sections. The number of spermatozoa within epididymal tubules was markedly reduced. White arrowheads indicate immature spermatogenic cells. (J) Immunofluorescence staining of the cauda epididymis. Hoechst was used to label nuclei (blue), acetylated tubulin to mark sperm flagella (green), and DDX4 to identify immature germ cells (magenta). White arrowheads indicate immature spermatogenic cells.

Given that FBXO39 contains an F‐box domain, we hypothesized that FBXO39 may interact with SKP1 to form the SCF complex in a manner similar to FBXO36. To test this possibility, we transiently expressed FBXO39 and SKP1 in HEK293T cells and found that wild‐type FBXO39 bound to SKP1 while mutant FBXO39 lacking the F‐box domain failed to bind (Figure 3A). These results suggest that FBXO39 can assemble into the SCF complex via its F‐box domain and may function as a component of an E3 ubiquitin ligase.

To investigate the function of *Fbxo39* in vivo, we generated *Fbxo39* KO mice using the CRISPR/Cas9 system, as described for *Fbxo36* KO mice. Two guide RNAs were designed to introduce a large deletion in the *Fbxo39* ORF (Figure 3B). The Cas9–sgRNA ribonucleoprotein complex was delivered into 64 zygotes by electroporation. Fifty‐six embryos were then transferred into the oviducts of 2 pseudopregnant females, and 22 pups were obtained. Genotyping PCR identified large deletions in 14 of these mice. The founder mouse was crossed with wild‐type mice, and subsequent intercrossing of heterozygous offspring resulted in the generation of KO mice (Figure 3C). Sanger sequencing verified a 2,325 bp deletion (Figure 3D).

Mating tests revealed that the number of pups per copulatory plug was significantly reduced in *Fbxo39* KO males compared to controls (Figure 3E), indicating that *Fbxo39* is required for normal male fertility. To investigate the basis of subfertility observed in *Fbxo39* KO mice, we first performed histological analyses of the reproductive system. Testis morphology and weights were indistinguishable between the two genotypes (Figure 3F, G). We next examined testicular and cauda epididymal sections by PAS staining. In WT testes, round spermatids undergo nuclear compaction, and elongating spermatids are evenly aligned along the lumen of seminiferous tubules. In contrast, in *Fbxo39* KO testes, incompletely compacted elongating spermatids were observed, along with a reduced number of elongating spermatids and the presence of abnormally retained elongated spermatids (Figures 3H and S3). PAS staining of the cauda epididymis further revealed a significant reduction in the number of mature spermatozoa, accompanied by the presence of abnormally transported immature spermatogenic cells, in *Fbxo39* KO mice (Figure 3I). Immunofluorescence analysis of cauda epididymal cross‐sections demonstrated that DDX4‐positive germ cells were aberrantly seen in the epididymal lumen of *Fbxo39* KO males (Figure 3J), indicating that immature spermatogenic cells were abnormally transported into the epididymis. Collectively, these findings indicate that *Fbxo39* deletion leads to impaired spermiogenesis and reduced male fertility in mice.

### 3.4 *Fbxo39* KO Spermatozoa Exhibit Abnormal Morphology and Impaired Motility

**FIGURE 4:**
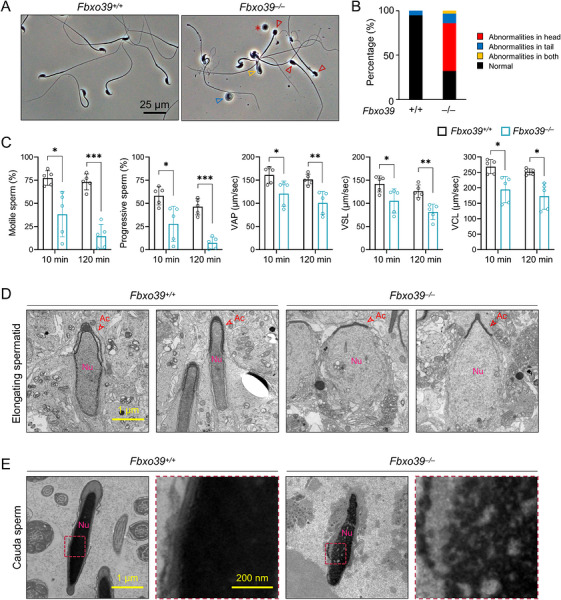
Evaluation of sperm morphology and velocity, and ultrastructural analysis of testes and cauda epididymis in *Fbxo39* KO mice. (A) Morphology of spermatozoa from the cauda epididymis of WT and *Fbxo39* KO males. Red arrowheads indicate abnormally shaped sperm heads, red asterisks indicate immature germ cells, yellow arrowheads indicate defective sperm tails, and blue arrowheads indicate coiled spermatozoa. (B) Quantification of abnormal sperm morphology in *Fbxo39* KO males. (C) Sperm motility analysis following incubation in capacitation medium. Percentages of motile and progressively motile spermatozoa were evaluated at 10 min and 120 min. Kinematic parameters, including VAP, VSL, and VCL, were also assessed at the same time points. (D) Elongating spermatids were examined by transmission electron microscopy (TEM). Nuclei (Nu) and acrosomes (Ac) are indicated. (E) Nuclei of cauda epididymal spermatozoa were examined by TEM.

To further evaluate the consequences of *Fbxo39* deletion, we analyzed spermatozoa from the cauda epididymis. WT spermatozoa exhibited a crescent‐shaped head and a long flagellum, whereas KO spermatozoa frequently displayed abnormal head morphology and/or disrupted flagella (Figure 4A, B). In addition, CASA analyses revealed impaired sperm motility in *Fbxo39* KO mice, with significantly reduced percentages of motile and progressively motile spermatozoa as well as decreased average path velocity (VAP), straight‐line velocity (VSL), and VCL at both 10 and 120 min of incubation in capacitation medium (Figure 4C). These findings indicate that *Fbxo39* deficiency affects both sperm morphology and motility.

### 3.5 Analysis Reveals Defective Head and Flagellar Morphology During Spermiogenesis in *Fbxo39* KO Testes

To further characterize defects in spermiogenesis caused by *Fbxo39* deletion, we performed transmission electron microscopy (TEM) on testes and cauda epididymis. During spermiogenesis, spermatid nuclei normally undergo condensation and elongation [30, 31, 32]. In step 8 round spermatids, no overt abnormalities were detected in acrosome or nuclear morphology between WT and KO mice (Figure S4A). In contrast, elongating and elongated spermatids in KO testes displayed defective nuclear condensation, abnormal head shaping, and incomplete acrosome formation (Figures 4D and S4B, C). Notably, abnormal elongating spermatids were observed in stage V–VI seminiferous tubules, where normally only elongated spermatids are present, indicating a delay or disruption in nuclear condensation (Figure S4B). This defective nuclear condensation may be associated with the abnormal acrosome formation observed in elongating spermatids (Figure 4D). Further, in step 16 spermatids, we observed a punctate electron‐dense pattern in KO sperm nuclei, suggesting altered nuclear condensation (Figure S4C). TEM analysis of cauda epididymal spermatozoa also revealed the punctate electron‐dense pattern in *Fbxo39* KO sperm nuclei (Figure 4E).

Examination of sperm flagellar structures also revealed abnormalities. TEM analysis of testicular spermatids showed defects in mitochondrial sheath formation in step 16 spermatids, while the axonemal structure appeared intact (Figure S4D). Further, in cauda epididymal spermatozoa, midpiece and axonemal defects were observed (Figure S4D). These abnormal flagellar structures may contribute to the reduced sperm motility observed in *Fbxo39* KO mice.

## 4 Discussion

In this study, we investigated the functions of two testis‐enriched F‐box proteins, FBXO36 and FBXO39, in mouse spermatogenesis. Both genes are highly conserved between mouse and human and display stage‐specific expression during germ cell development. Phylogenetic analysis revealed that FBXO36 and FBXO39 are evolutionarily distinct members of the FBXO family (Figure S1A), indicating that they may have different functional contributions. Consistently, while *Fbxo36* was dispensable for male fertility, *Fbxo39* was found to be important for spermiogenesis. These findings underscore the functional divergence within the FBXO family and identify *Fbxo39* as a critical regulator of male reproduction.

Although *Fbxo36* disruption did not cause marked defects in spermatogenesis or male fertility, a mild reduction in the sperm velocity parameter, VCL, was detected, which was insufficient to impair in vivo fertility. This mild phenotype could be because of functional redundancy with other FBXO proteins, such as FBXO30, which is closely related in the phylogenetic analysis (Figure S1A). Alternatively, given that F‐box proteins mediate proteasomal degradation, FBXO36 may regulate the amounts of specific proteins associated with sperm motility, which may be critical under conditions not examined in this study.

In contrast, FBXO39 plays a crucial role in maintaining normal male fertility. *Fbxo39* KO males exhibited reduced litter size, abnormal sperm morphology, and impaired sperm motility. Histological and ultrastructural analyses revealed defective sperm head shaping and altered nuclear condensation during spermiogenesis, possibly leading to abnormal retention of immature spermatids in the epididymis (Figure 3H). In addition to head abnormalities, TEM analysis revealed structural defects in the mitochondrial sheath of the flagellum in both testes and cauda epididymis, while axonemal abnormalities were observed in cauda epididymal spermatozoa (Figure S4D). These findings raise the possibility that defects in mitochondrial sheath formation may destabilize axonemal structure during epididymal transit, leading to the axonemal abnormalities observed in cauda epididymal spermatozoa. Although mitochondrial sheath defects were detected in the testis, abnormalities in nuclear condensation were observed at an earlier stage of spermiogenesis, suggesting that head malformation represents a primary defect. Given the close coordination between sperm head shaping and mitochondrial sheath assembly [33], the mitochondrial abnormalities may arise secondarily to impaired head morphogenesis. However, it also remains possible that FBXO39 independently contributes to both nuclear condensation and mitochondrial sheath formation during spermiogenesis.

SKP1 (S‐phase kinase–associated protein 1) is a central component of the SCF E3 ubiquitin ligase complex and is expressed in meiotic germ cells, where it localizes to the synaptonemal complex and is essential for meiotic progression [34]. In addition, FBXO47, another testis‐enriched F‐box protein, regulates meiosis by targeting HORMAD1 for degradation via the ubiquitin–proteasome pathway [35], illustrating how testis‐enriched F‐box proteins can mediate stage‐specific protein turnover. FBXO39 interacts with SKP1 via its F‐box domain (Figure 3A), suggesting a role as a substrate‐recognition subunit of the SCF complex. This interaction may contribute to ubiquitin‐mediated regulation of proteins required for proper sperm head maturation, although FBXO39 may also have SCF‐independent roles. Identification of specific SCF substrates will be critical for understanding the spermiogenic defects in *Fbxo39* KO mice.

**FIGURE 5:**
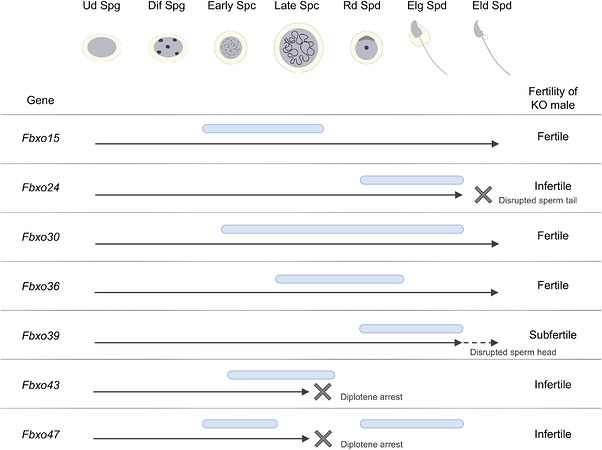
Summary of KO mouse phenotypes for testis‐enriched FBXO family. Expression data of each gene in mouse germ cells were obtained from a published testis scRNA‐seq dataset [22]. Expression levels were normalized by Z‐score, with values >0.4 indicated as blue bars. KO studies of testis‐enriched FBXO proteins have revealed diverse roles in male germ cell development. Black arrows denote the stage of spermatogenesis affected in KO mice. Cell types are abbreviated as follows: undifferentiated spermatogonia (Ud Spg); differentiating spermatogonia (Dif Spg); early spermatocytes (Early Spc); late spermatocytes (late Spc); round spermatids (Rd Spd); elongating spermatids (Elg Spd); elongated spermatids (Eld Spd).

By analyzing FBXO36 and FBXO39 in this study, KO mouse phenotypes of all testis‐enriched FBXOs were elucidated. Our findings align with previous reports indicating that several testis‐enriched FBXO proteins are individually important for male germ cell development, including FBXO24 in spermiogenesis [10, 11, 12] and FBXO43 and FBXO47 in meiosis [13, 14]. These studies emphasize the distinct and non‐redundant roles of some FBXO proteins in spermatogenesis, possibly by recognizing different substrates (Figure 5). The identification of FBXO39 as a key regulator of spermiogenesis further expands the repertoire of FBXO proteins implicated in male reproduction.

The conservation of the *Fbxo39* expression pattern between mouse and human testes suggests potential clinical relevance. A substantial proportion of male infertility cases remain idiopathic [36], and abnormalities in genes associated with spermiogenesis are likely underrecognized [37, 38]. Considering reduced male fertility observed in *Fbxo39* KO mice, mutations or dysregulation of FBXO39 may represent an unrecognized cause of human male infertility, particularly in cases presenting with teratozoospermia and/or asthenozoospermia. According to the Genome Aggregation Database (gnomAD), several loss‐of‐function variants in *FBXO39*, including stop‐gained and frameshift mutations, have been identified at appreciable frequencies (e.g., p.Trp141Ter; allele frequency: 2.54 × 10^–^^5^) (Figure S5). These observations raise the possibility that *FBXO39* variants contribute to impaired spermiogenesis and male infertility in humans. Future investigations of FBXO39 variants in infertile men, combined with functional analyses in model systems, will be essential to establish FBXO39 as a candidate gene for the clinical diagnosis of male infertility.

## Author Contributions

The study was designed by Y.K., H.M., and M.I. Data were collected, analyzed, and interpreted by all authors. The manuscript was drafted by Y.K. and revised by H.M. and M.I.

## Conflicts of Interest

The authors declare they have no conflicts of interest.

## Supporting information




**Supporting File 1**: andr70225‐sup‐0001‐SupMat.pdf
