# Supplementary material for: Testis‐Enriched F‐Box Protein FBXO39 Is Important for Spermiogenesis and Male Fertility in Mice
Source: Andrology. 2026 Mar 31;14(6):1636–46. doi: 10.1111/andr.70225 (PMC13432620; doi:10.1111/andr.70225)
Supplement: Supplementary file 1 — Supporting File 1: andr70225‐sup‐0001‐SupMat.pdf [file ANDR-14-1636-s001.pdf]

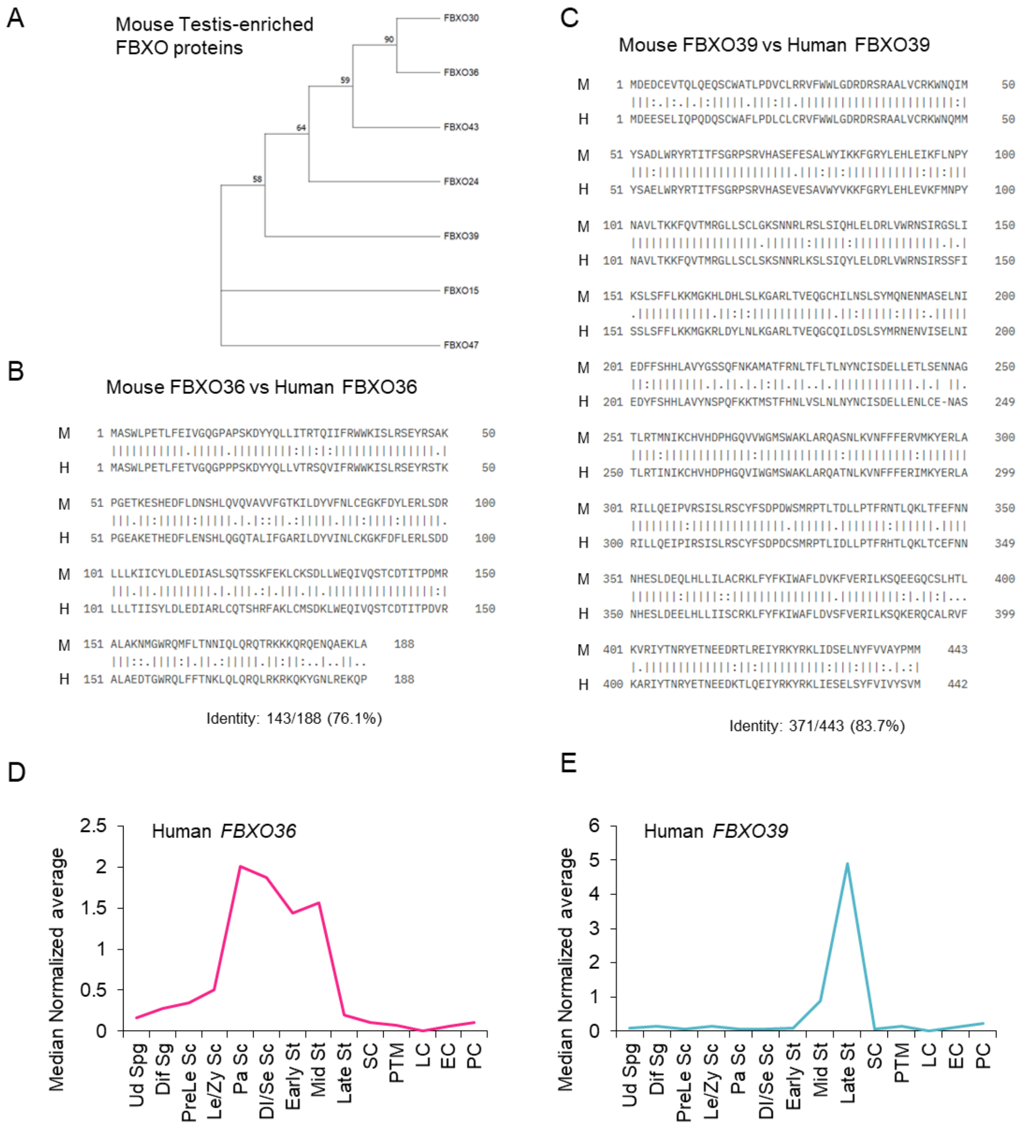

Figure S1. Amino acid sequence comparison and expression patterns.

(A) Phylogenetic analysis of testis-enriched FBXO paralogs in mice. Bootstrap values (percentages of 500 replicates) are indicated next to the branches. (B) Pairwise alignment of mouse and human FBXO36 amino acid sequences using EMBOSS Needle (https://www.ebi.ac.uk/jdispatcher/psa/emboss_needle). M: Mouse; H; Human. (C) Pairwise alignment of mouse and human FBXO39 amino acid sequences using EMBOSS Needle. (D) Expression of *FBXO36* across cell types in human testes. Dif Spg: differentiating spermatogonia. PC: perivascular cells. (E) Expression of *FBXO39* across cell types in human testes.

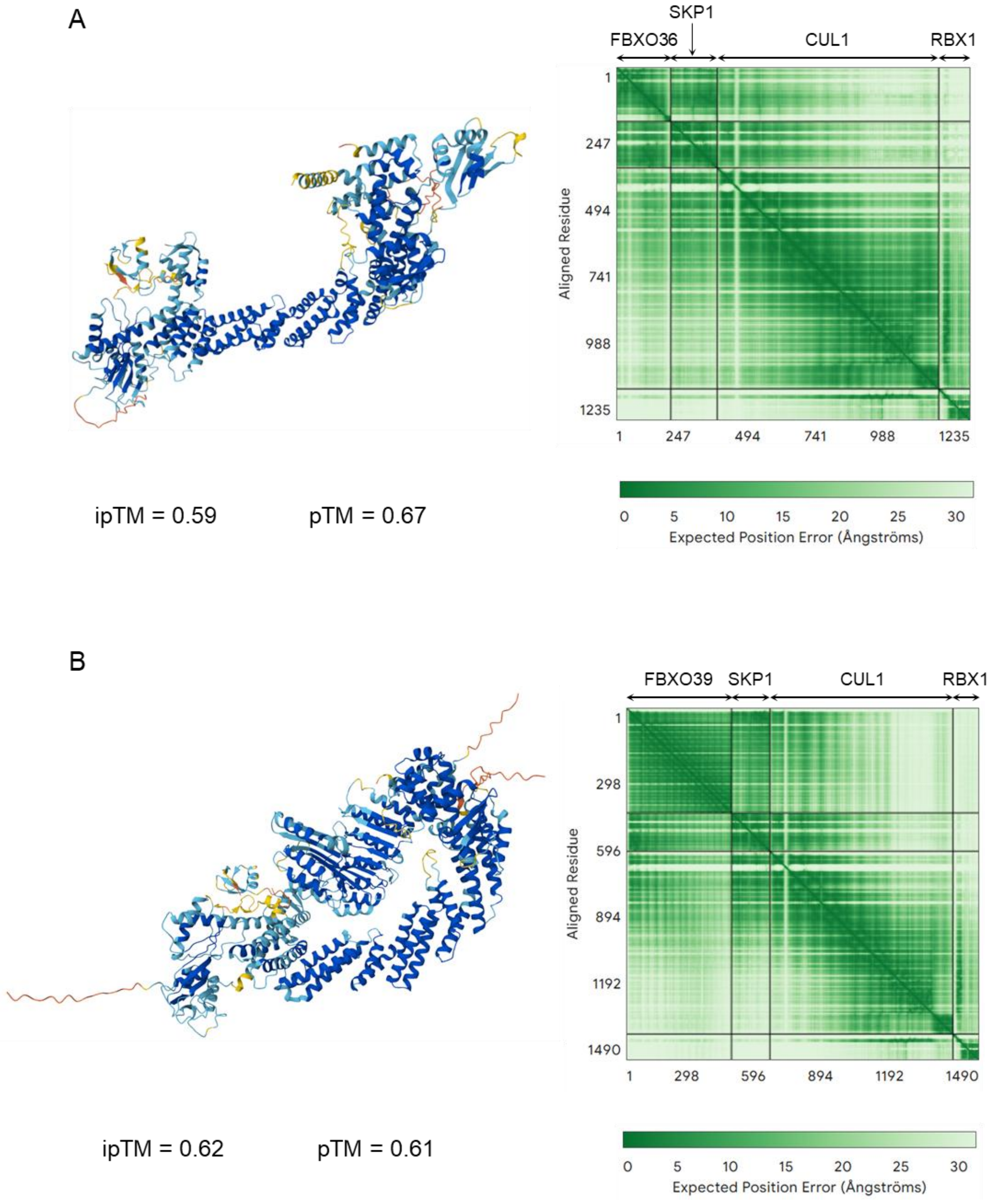

Figure S2. Predicted structures of mouse SCF complexes generated by AlphaFold3.

(A) Predicted structure of the mouse SCF (FBXO36) complex obtained using AlphaFold3. The model is color-coded according to the pLDDT score. The right panel shows the predicted aligned error (PAE) plot. iPTM and pTM scores are also indicated. (B) Predicted structure of the mouse SCF (FBXO39) complex obtained using AlphaFold3.

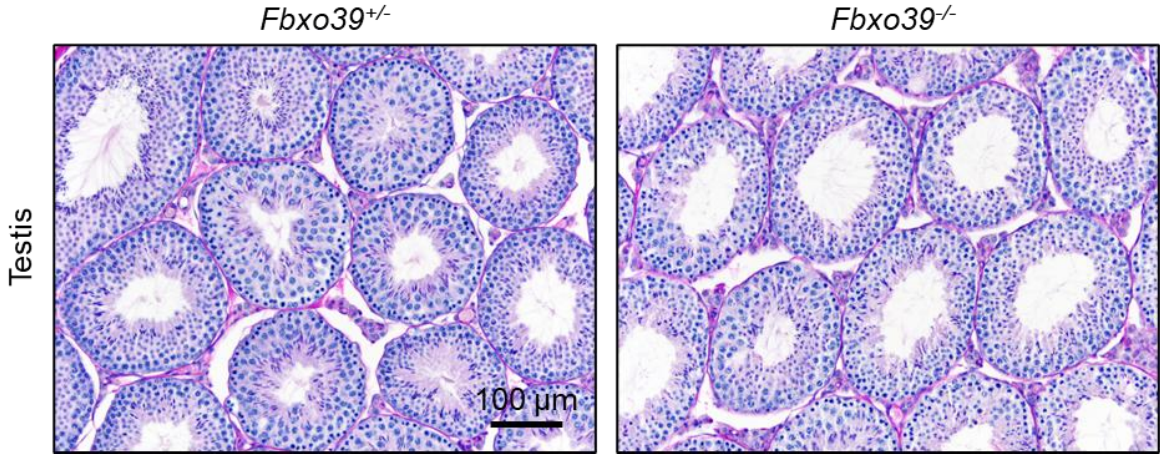

Figure S3. PAS and hematoxylin staining of testis sections.

Low-magnification views of testis sections from *Fbxo39* heterozygous and KO mice are shown.

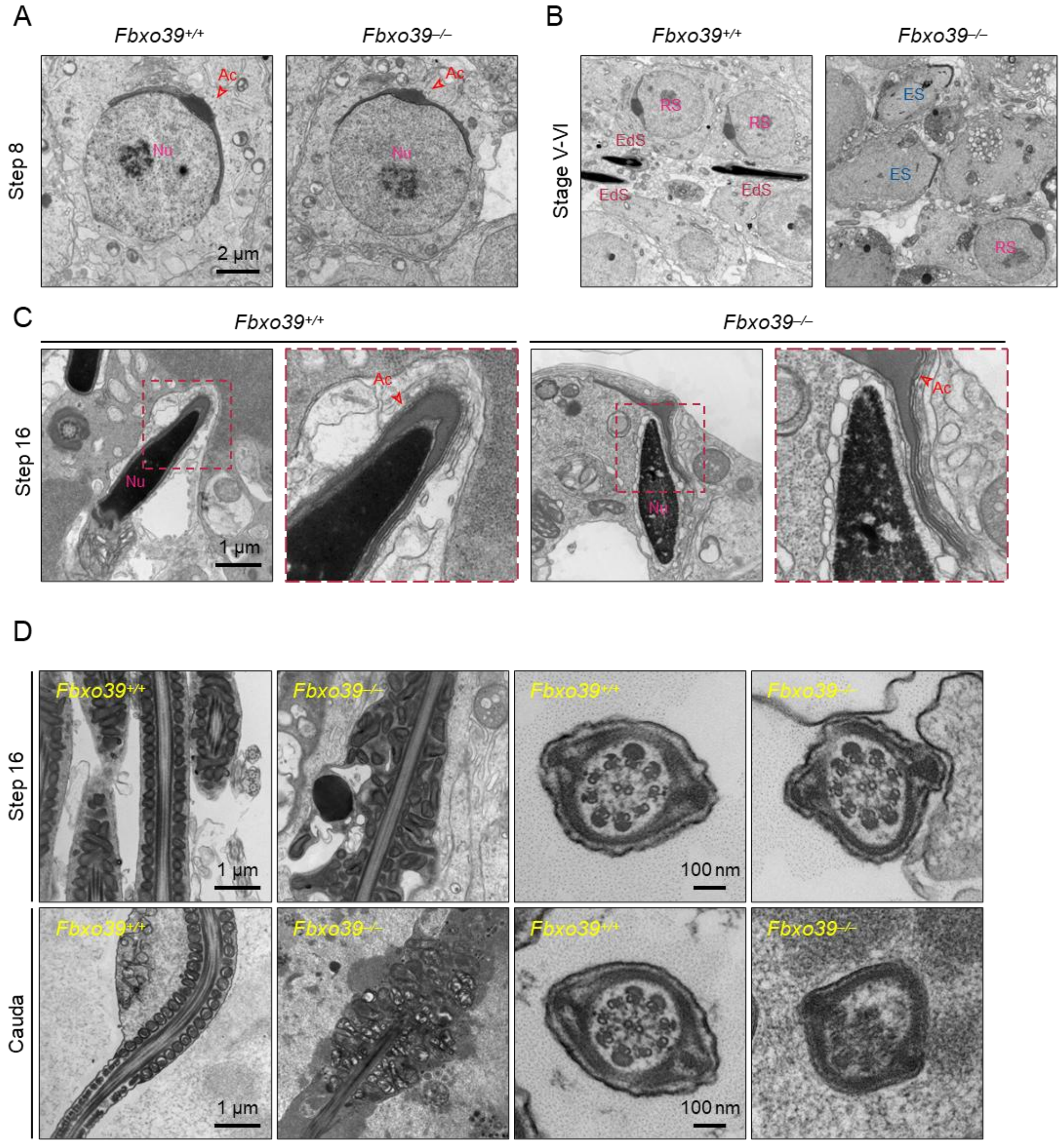

Figure S4. TEM analysis of testes and cauda epididymis in *Fbxo39* KO mice.

(A) Step8 round spermatids were examined by transmission electron microscopy (TEM). Nuclei (Nu) and acrosomes (Ac) are indicated. (B) Stage V–VI seminiferous tubules were examined. Round spermatids (RS), elongating spermatids (ES), and elongated spermatids (EdS) are labeled. (C) Step 16 elongated spermatids in the testis were examined. (D) Flagella of step 16 spermatids and cauda epididymal spermatozoa were analyzed.

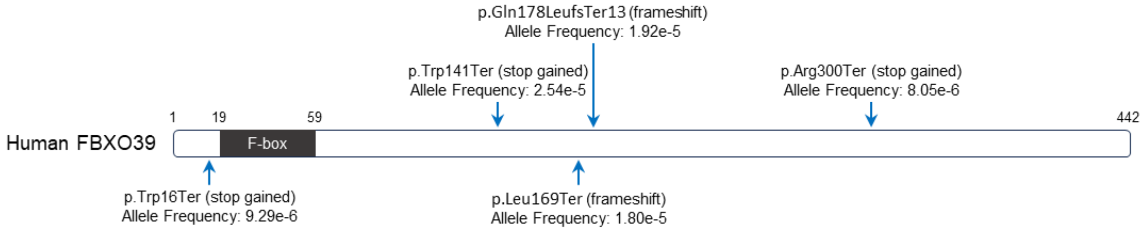

Figure S5. Human *FBXO39* variants.

The five most common loss-of-function (LoF) mutations reported in gnomAD are shown, including frameshift and stop-gained variants. The F-box domain was annotated using SMART.

Table S1. Primers and gRNAs used in this study.

Method	Symbol	Sequence (5'→3')
RT-PCR/Cloning	*Fbxo36* Fw	atgaattcatggcgtcgtggctgccggag
	Fbxo36 Rv	gcgtcgacagcaagcttctctgcctggt
	Fbxo39 Fw	atgatatcgccgccatggatgaagactgtgaagtgacc
	Fbxo39 Rv	gcgctagccatcatggggtaggcgacgac
	Actb Fw	catccgtaaagacctctatgccaac
	Actb Rv	atggagccaccgatccaca
Genotyping	*Fbxo36* KO Fw (Fw #1)	actatgcctgccaacacttga
	Fbxo36 KO Rv (Rv #1)	acaagtgcctctttcctggg
	Fbxo36 WT Fw (Fw #2)	gcactgggctcactcatacc
	Fbxo36 WT Rv (Rv #2)	ggtcagtgggacaaagcaag
	Fbxo39 KO Fw (Fw #3)	cagacagcttctcactatgc
	Fbxo39 KO Rv (Rv #3)	gaaaaaggaatgagggcacg
	Fbxo39 WT Fw (Fw #4)	aaatcacggtacactggctg
	Fbxo39 WT Rv (Rv #4)	aagcagctgagagtccttacc
iPCR	*Fbxo36* iPCR #1	gtccagtccacctgtgacaccatc
	Fbxo36 iPCR #2	ccgctccaggtagtcaaatttacc
	Fbxo39 iPCR #1	agtggcccagcagctctgctc
	Fbxo39 iPCR #2	accatcacgttcagcggaagg
gRNA	*Fbxo36* gRNA #1	tacagatcatctttagatgg
	Fbxo36 gRNA #2	ccacagtggtccgaatctcc
	Fbxo39 gRNA #3	caccactacgctgtacatca
	Fbxo39 gRNA #4	atttcctgtaaatttcccgt

Table S2. Antibodies used in this study.

Antibodies	Host	Source	#	Method
anti-Acetylated Tubulin	Mouse	SIGMA	T7451	IF
anti-β-actin	Rabbit	MBL	PM053	IB
anti-DDX4	Rabbit	abcam	ab13840	IF
anti-FLAG M2	Mouse	SIGMA	F1804	IB
anti-DDDDK-tag pAb	Rabbit	MBL	PM020	IB
anti-1D4 tag	Mouse	A kind gift from Dr. Martin M. Matzuk		IB
anti-rabbit IgG-HRP	Goat	Jackson ImmunoResearch	#111-036-045	IB
anti-mouse IgG-HRP	Goat	Jackson ImmunoResearch	#115-036-062	IB
anti-rabbit IgG-Alexa Fluor 546	Goat	Thermo Fisher Scientific	A-11071	IF
anti-mouse IgG-Alexa Fluor 488 Highly Cross-Adsorbed	Goat	Thermo Fisher Scientific	A-11029	IF

IB: Immunoblotting
IF: Immunocluorescence
